# Hypermethylation of APC2 Is a Predictive Epigenetic Biomarker for Chinese Colorectal Cancer

**DOI:** 10.1155/2018/8619462

**Published:** 2018-10-29

**Authors:** Yuan He, Li-Yue Sun, Jing Wang, Rui Gong, Qiong Shao, Zi-Chen Zhang, Zu-Lu Ye, Hai-Yun Wang, Rui-Hua Xu, Jian-Yong Shao

**Affiliations:** ^1^State Key Laboratory of Oncology in South China, Collaborative Innovation Center for Cancer Medicine, Sun Yat-sen University Cancer Center, Guangzhou 510060, China; ^2^Department of Molecular Diagnostics, Sun Yat-sen University Cancer Center, Guangzhou 510060, China; ^3^Department of Chemical Medicine, Sun Yat-sen University Cancer Center, Guangzhou 510060, China; ^4^School of Laboratory Medicine, Wannan Medical College, Wuhu 241002, China

## Abstract

**Objective:**

To investigate methylation of the adenomatosis polyposis coli homologue (APC2) promoter and its correlation with prognostic implications in Chinese colorectal cancer (CRC).

**Methods:**

The mRNA expression of APC2 in colorectal tissues was evaluated using the database of The Cancer Genome Atlas (TCGA). Methylation analysis of APC2 in tumor (*n* = 66) and corresponding adjacent formalin-fixed and paraffin-embedded (FFPE) tissues (*n* = 44) was performed by Sequenom EpiTYPER® and verified by cloning-based bisulfite sequencing analysis. Demethylation and retrieval of APC2 expression in cell lines HT29, HCT116, and SW480 were treated with 5-aza-2′-deoxycytidine (5-AZC).

**Results:**

Analysis of TCGA showed that APC2 mRNA was significantly downregulated in primary tumors when compared to normal tissues (*p* < 0.05). APC2 methylation was upregulated (43.93% vs 7.31%, *p* < 0.05) in tumors compared to adjacent FFPE tissues. In vitro experiments demonstrated that 5-AZC downregulated the methylation of APC2 and retrieved its expression of mRNA and protein levels (*p* < 0.05). Multivariate Cox regression indicated that APC2_CPG_14 was an independent risk factor for overall survival (HR = 6.38, 95% CI: 1.59–25.64, *p* < 0.05).

**Conclusion:**

This study indicates that APC2 is hypermethylated and may be a tumorigenesis biomarker for Chinese CRC patients.

## 1. Introduction

Colorectal cancer (CRC) is one of the leading causes of cancer-related mortality with high incidence worldwide [1]. Despite advances in efficient surgical techniques and novel chemoradiotherapeutic interventions, the long-term survival rate of CRC patients remains poor [2]. Moreover, there are often challenging cases in which the patients' survival is inconsistent. There is a need to identify novel biomarkers for targeted therapy. Recently, promising epigenetic therapies for cancer have been put forward [3].

Methylation of CpG sites is a common epigenetic modification in cancers and results in gene silencing and oncogenesis [4, 5]. The classic tumor suppressor gene adenomatosis polyposis coli (APC) has been well studied in many malignancies [6–8]. Its homologue gene, APC2/APCL, located on chromosome 19p13.3 [9], plays a significant role in several human cancers, including retinoblastoma (RB) tumor, lymphocytic leukemia, and ovarian cancer [10–13]. Endogenous APC2 is diffusely located in the cytoplasm where it couples the Golgi apparatus and actin filaments together [14]. Previous functional studies have shown it to be involved in microtubule tethering [15] and that it affects cell cycle progression by interacting with EB1 [16]. APC2 interacts with cytoplasmic beta-catenin (*β*-catenin) and regulates the Wnt signaling pathway [17]. Furthermore, APC2 has been found to be hypermethylated in both RB tumor samples and the Y79 cell line, mediating the reduction of *β*-catenin levels [10]. However, its function upon methylation in Chinese CRC has not been reported previously. Here, we aimed to investigate the role and clinical significance of APC2 methylation in Chinese CRC.

## 2. Materials and Methods

### 2.1. Bioinformative Analysis of Oncomine

The mRNA expression of APC2 in a number of CRC tissues was evaluated by The Cancer Genome Atlas (TCGA) in Oncomine (https://www.oncomine.org), a database of RNA and DNA sequencing information originated from the Gene Expression Omnibus, The Cancer Genome Atlas, and other published literature database. “APC2,” “Cancer vs Normal Analysis,” “Colorectal cancer,” and “mRNA” were used as search terms to obtain the expression data in CRC. The data was presented as log^2^ median-centered intensity in the microarray datasets.

### 2.2. CRC Clinical Samples and Cell Lines

A total of 66 tumor and 44 corresponding adjacent formalin-fixed and paraffin-embedded (FFPE) tissues were obtained from the Sun Yat-sen University Cancer Center (SYSUCC), Guangzhou, China, between 2013 and 2017. The tissues were selected and confirmed histologically based on the following criteria: primary and curative resection of the tumor with an availability of follow-up data and no preoperative anticancer treatment. Human CRC cell lines (HT29, HCT116, and SW480) were obtained from the Chinese Academy of Sciences, Shanghai Institutes for Cell Resource Center. HT29 and HCT116 were cultured in McCoy's 5A with L-glutamine (HyClone, Logan, UT, USA), and SW480 was maintained in incomplete Leibovitz's L-15 medium (KeyGEN, Nanjing, Jiangsu, China) media supplemented with 10% heat-inactivated fetal bovine serum (Gibco, Carlsbad, CA, USA), respectively. Cells were grown in a humidified incubator at 37°C with 5% CO_2_. The study was approved by the Ethics Committee of SYSUCC.

### 2.3. DNA Isolation and Sequenom EpiTYPER® Analysis

Genomic DNA was isolated from the tissue samples and cell lines using a nucleic acid isolation kit (Qiagen, Hilden, NW, Germany). Subsequently, DNA extracted from each specimen was subjected to bisulfite treatment using the EZ conversion kit (Zymo Research, Orange, CA, USA) according to the manufacturer's instructions. Methylation of APC2 was quantitatively analyzed using the Sequenom MassArray platform (Agena Bioscience, San Diego, CA, USA), which employs matrix-assisted laser-desorption/ionization time-of-flight mass spectrometry and RNA base-specific cleavage. An APC2 sense primer 5′-ATTGAGGTTGGGGGGTGTAG-3′ and antisense primer 5′-AAAATAAAAAAAAATAAAATAAAATAAAC-3′ were used. Each forward and reverse primer was modified with a 10-mer tag (5′-AGGAAGAGAG-3′) or a T7-promoter tag (5′-CAGTAATACGACTCACTATAGGGAGAAGGCT-3′) for transcription in vitro. Altogether, 25 CpG sites in this region were checked. The spectra methylation ratios were generated on EpiTYPER 1.0 (Agena Bioscience, San Diego, CA, USA).

### 2.4. Bisulfite-Specific PCR (BSP) and Sequencing

The bisulfite-treated DNA was amplified by PCR for APC2 gene with bisulfite-specific PCR (BSP) specific primer pair (forward: 5′-ATTGAGGTTGGGGGGTGTAG-3′; reverse: 5′-AAAATAAAAAAAAATAAAATAAAATAAAC-3′) under the following conditions: 94°C denaturation for 3 min, 40 cycles of 94°C for 30 s, 50°C for 30 s, 72°C for 30 s, and 72°C elongation for 5 min. The PCR products were separated by electrophoresis in 1.5% agarose gel with ethidium bromide. Bands were isolated from the gel and purified with Wizard SV Gel and PCR Clean-Up System (Tiangen, Beijing, China). Following this, the purified PCR products were subcloned into a TOPO Vector (Ruibo, Guangzhou, Guangdong, China) according to the manufacturer's instructions. Positive clones were obtained by ampicillin selection followed by PCR colony screening. Eight positive clones were randomly selected for sequencing at Ruibo (Ruibo, Guangzhou, Guangdong, China).

### 2.5. Demethylation by 5-Aza-2′-Deoxycytidine (5-AZC) Treatment

For validation of the role of epigenetics in transcriptional regulation, HT29, HCT116, and SW480 were used to conduct an in vitro functional study of APC2 on 6 well plates. Briefly, 1 × 10^5^ cells were incubated with demethylating agent 5-AZC (0 *μ*mol/L vs 5 *μ*mol/L vs 10 *μ*mol/L) and with media changed every 24 hours for three days.

### 2.6. RNA Extraction and qRT-PCR

TRIzol (Invitrogen, Carlsbad, CA, USA) was used to purify the total RNA of cell lines. Reverse transcription and qRT-PCR were performed using a SYBR Green qRT-PCR kit (Takara, Shiga, Japan) and CFX96 real-time polymerase chain reaction (PCR) detection system (Bio-Rad, Hercules, CA, USA) in triplicates. GAPDH was used as an internal reference. The primers used were synthesized by Ruibo (Ruibo, Guangzhou, Guangdong, China) and were as follows: APC2 (forward: 5′-CTGTACCGGGTCTCTGCAGTGTTA-3′ and reverse: 5′-TACGCCGGACAGATGGCTTTA-3′) and GAPDH (forward: 5′-GCACCGTCAAGGCTGAGAAC-3′ and reverse: 5′-TGGTGAAGACGCCAGTGGA-3′).

### 2.7. Immunocytochemistry (ICC)

In sum, 1 × 10^4^ cells (HT29, HCT116, and SW480) were seeded on 24-well plates and incubated for 72 hours. After being washed with PBS, the cells were fixed with 10% paraformaldehyde at room temperature for 10 min. Then, they were incubated with a blocking solution (10% fetal bovine serum in PBS) for 10 min followed by anti-rabbit APC2 polyclonal antibody (1 : 500, Sigma, St. Louis, MO, USA) for 1 hour at room temperature. Afterwards, cells were washed and displayed using a GTVision™ III Detection System (GK5007, Copenhagen, Denmark). Staining with PBS instead of primary antibody against APC2 was used as negative control. Experiments were repeated in triplicates.

### 2.8. Statistical Analysis

We used SPSS 20.0 (IBM Corporation, Armonk, NY) to perform statistical analyses. Student's *t*-test, an ANOVA test, and *χ*^2^ test were utilized to analyze the categorical or continuous variables, respectively. In addition, Spearman's correlation was used to compare the linear association between the methylation and transcription level. The multivariate Cox regression model was used to assess the potential independent prognostic factors. All tests were two sided at a significance level of 0.05.

## 3. Results

### 3.1. Bioinformative Evaluation of APC2 mRNA Expression

**Figure 1:**
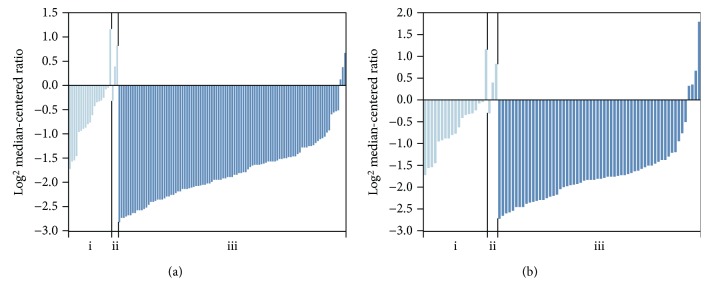
Analysis of APC2 mRNA expression in TCGA (fold change ≥ 2, *p* < 0.0001). (a) The APC2 mRNA expression of the (i) colon, (ii) rectum, and (iii) colon adenocarcinoma (101 patients). (b) The APC2 mRNA expression of the (i) colon, (ii) rectum, and (iii) rectal adenocarcinoma (60 patients).

The mRNA expression of APC2 in CRC was explored in TCGA database. As depicted in Figure 1, the mRNA expression of APC2 was found to be significantly downregulated in colorectal adenocarcinoma compared to normal tissues (fold change ≥ 2, *p* < 0.0001).

### 3.2. The Methylation Expression of APC2 and Its Clinicopathological Features in CRC Tissues

**Figure 2:**
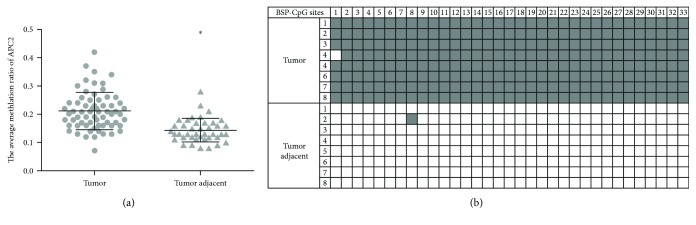
(a) Sequenom EpiTYPER system analysis of the average methylation ratio of APC2 in CRC tissues, ^∗^*p* < 0.05. (b) Representative APC2 methylation levels between the tumor and adjacent tissue samples using BSP PCR-based sequencing analysis. Methylation levels in the tumor samples were higher than in the adjacent samples. Each row represents an individually sequenced clone and each column square, a CpG residue. White and black squares represent unmethylated and methylated cytosines, respectively (*p* < 0.05).

To investigate the potential biological function of APC2 methylation, 66 CRC tissues and 41 corresponding adjacent tissues were included from SYSUCC. Their clinicopathological features are summarized in Table 1. APC2 was significantly hypermethylated in tumor compared to adjacent tissues (43.93% vs 7.31%, *p* < 0.05, Figure 2). Meanwhile, representative results for BSP were consistent with the result described above (Figure 2). Additionally, female patients tended to have significantly increased APC2 hypermethylation compared to males (*p* < 0.05). There was no significant correlation between APC2 methylation and other clinicopathological parameters, such as age, performance status, tumor location, pathological differentiation, TNM stage, CEA, Ki67, or fecal occult blood (*p* > 0.05).

### 3.3. Hypermethylation of APC2 and Downregulation of Transcription Levels in CRC Cell Lines

**Figure 3:**
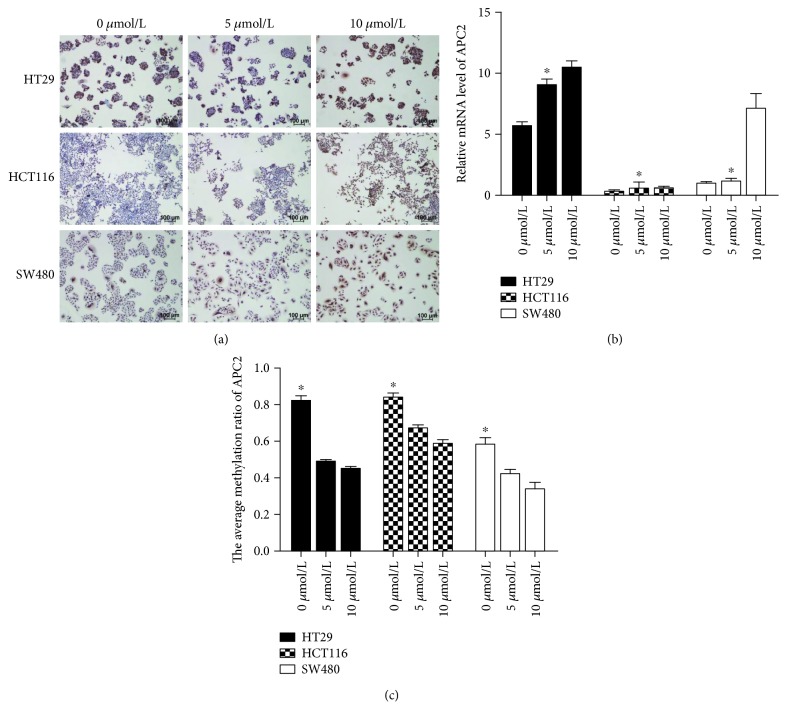
(a) ICC staining with APC2 expression in the membrane of CRC cell lines. (b) SYBR analysis of the relative mRNA level of APC2 in CRC cell lines, ^∗^*p* < 0.05. (c) Sequenom EpiTYPER system analysis of the average methylation ratio of APC2 in CRC cell lines, ^∗^*p* < 0.05.

Then, we investigated APC2 methylation levels in HT29, HCT116, and SW480 cell lines. To confirm that the downregulation of transcription levels in cell lines was attributed to promoter hypermethylation, we treated cells with 5-AZC (0 *μ*mol/L vs 5 *μ*mol/L vs 10 *μ*mol/L). The Sequenom MassArray results showed a gradual but clear reduction in APC2 methylation expression over 72 h following treatment (*p* < 0.05, Figure 3). Meanwhile, APC2 mRNA and protein were found to gradually but significantly increase (*p* < 0.05, Figure 3). These data suggest that APC2 hypermethylation may be responsible for the downregulation of mRNA and protein expression in CRC.

### 3.4. The Relationship between APC2 Methylation and Overall Survival

To explore the overall survival implications for APC2 in CRC, a multivariate regression analysis based on the Cox proportional hazard model was used to test the independent risk factors. The results showed that APC2_CPG_14 was an independent prognostic factor for poor overall survival (HR = 6.38, 95% CI: 1.59–25.64, *p* = 0.00), as well as performance status (HR = 3.95, 95% CI: 1.51–10.29, *p* = 0.00) and Ki67 (HR = 0.28, 95% CI: 0.10–0.77, *p* = 0.01) (Table 2).

## 4. Discussion

CRC is a highly heterogeneous disease with poor clinical outcomes [18]. Previous studies suggested that methylation biomarkers could be used for the molecular characterization of various carcinomas, with implications for diagnosis and prognosis [3]. However, the possible molecular mechanism of APC2 inactivation or epigenetic silencing in CRC is unknown. Here, we report that APC2 was hypermethylated in CRC tissues and cell lines. An inverse correlation was observed between the APC2 methylation and its transcriptional levels. Furthermore, APC2_CPG_14 was an independent risk factor for overall survival in CRC patients.

Beta et al. [10] observed that 70% of RB tumors tested positive for APC2 methylation using methylation-specific PCR. Hypermethylation of the APC2 gene was also reported in a large-scale methylation analysis of lymphocytic leukemia [11]. Moreover, scholars using methylation-specific PCR (MSP) found that APC2 was hypermethylated in CRC patients [13, 19]. Our findings were generally in line with these previous studies, suggesting that APC2 hypermethylation might be a common occurrence in the development of CRC. The differences in positive rates of methylation may be attributed to the small sample size, to the ethnicities (Chinese vs Iranians + African-American) and to the different methods used. In contrast to MSP, Sequenom EpiTYPER used in this study was a mass spectrometry-based bisulfite sequencing method that was region-specific in a quantitative and high-throughput fashion. It targeted genomic regions of 100–600 base pairs and resulted in quantitative DNA methylation measurement at single-nucleotide resolution [20]. A greater number of CpG residues were analyzed for BSP across the whole amplicon when compared with MSP [21]. Furthermore, we found that APC2 methylation was significantly related with sex. It has been reported that methylated loci may differ and that the clinical role of concurrent methylation may be different in males and females with CRC [22, 23], suggesting that aberrant methylation of APC2 might be associated with patient sex.

Several studies have been carried out to explore the molecular mechanism of APC2 in cancers, and which imply that APC2 plays an important role in tumorigenesis and cancer development. One study indicated that APC2 was involved in actin-associated events, influencing cell motility or adhesion through actin filaments, as well as functioning independently or in cooperation with APC to downregulate *β*-catenin signaling [14]. APC2 was found to be an essential mediator of cytoskeletal regulation in response to extracellular signals [24]. Nakagawa et al. [25] suggested that APC2 might be involved in the p53/Bcl2-linked pathway of cell-cycle progression and cell death. Additionally, APC2 was found to be involved in other functions, including cell cycle progression, by interacting with EB1, microtubules, and Hdlg [15, 16]. For instance, the functional complementation of APC2 constituted a substantial facet of tumor development for the retention of 15R to target *β*-catenin in familial adenomatous polyposis (FAP) patients [26]. APC2 was repressed by miR-939 through the Wnt/*β*-catenin pathway in ovarian cancer [12]. Furthermore, the reduction of APC2 in RB led to increases in the Wnt signaling pathway protein *β*-catenin through methylation [10], which was in line with our study suggesting that the absence of APC2 in CRC was related to methylation mechanism.

Some limitations of this study need to be acknowledged. Firstly, the number of CRC patients in the study was limited, and the findings should therefore be interpreted with caution. Moreover, the detailed carcinogenetic mechanism was not elucidated in CRC, and this needs to be explored further.

## 5. Conclusions

In conclusion, the present study indicates that APC2 is hypermethylated and may serve as a tumorigenesis biomarker for Chinese CRC patients.

## Figures and Tables

**Table 1:** Relationship between APC2 methylation and clinicopathological parameters in CRC.

Clinical parameters	N (N1%)	Average methylation		
		<0.2	≥0.2	*p*
Sex				
Male	39 (59.1%)	26 (66.7%)	13 (33.3%)	0.037
Female	27 (40.9%)	11 (40.7%)	16 (59.3%)	
Age (years)				
<54	35 (53.0%)	19 (54.3%)	16 (45.7%)	0.758
≥54	31 (47.0%)	18 (58.1%)	13 (41.9%)	
Performance status				
0	2 (3.0%)	1 (50.0%)	1 (50.0%)	0.873
1	46 (69.7%)	25 (54.3%)	21 (45.7%)	
2	18 (27.3%)	11 (61.1%)	7 (38.9%)	
Tumor location				
Ascending	17 (25.8%)	8 (47.1%)	9 (52.9%)	0.877
Transverse	6 (9.1%)	3 (50.0%)	3 (50.0%)	
Descending	11 (16.7%)	6 (54.5%)	5 (45.5%)	
Sigmoid	19 (28.8%)	12 (63.2%)	7 (36.8%)	
Rectum	13 (19.7%)	8 (61.5%)	5 (38.5%)	
Differentiation				
High	0 (0.0%)	0 (0.0%)	0 (0.0%)	0.277
Moderate	36 (54.5%)	18 (50.0%)	18 (50.0%)	
Low	30 (45.5%)	19 (63.3%)	11 (36.7%)	
TNM				
I	2 (3.0%)	1 (50.0%)	1 (50.0%)	0.681
II	14 (21.2%)	9 (64.3%)	5 (35.7%)	
III	16 (24.2%)	7 (43.7%)	9 (56.3%)	
IV	34 (51.5%)	20 (58.8%)	14 (41.2%)	
T				
1	2 (3.0%)	0 (0.0%)	2 (100.0%)	0.132
2	1 (1.5%)	1 (100.0%)	0 (0.0%)	
3	36 (54.5%)	17 (47.2%)	19 (52.8%)	
4	27 (41.0%)	18 (66.7%)	9 (33.3%)	
N				
0	21 (31.8%)	10 (47.6%)	11 (52.4%)	0.716
1	25 (37.9%)	14 (56.0%)	11 (44.0%)	
2	20 (30.3%)	12 (60.0%)	8 (40.0%)	
M				
0	32 (48.5%)	16 (50.0%)	16 (50.0%)	0.472
1	34 (51.5%)	20 (58.8%)	14 (41.2%)	
CEA (ng/ml)				
0–4.9	21 (31.8%)	12 (57.1%)	9 (42.9%)	0.794
≥5	41 (62.1%)	22 (53.6%)	19 (46.4%)	
NG	4 (6.1%)			
Ki67				
0–59	20 (30.3%)	9 (45.0%)	11 (55.0%)	0.308
≥60	39 (59.1%)	23 (59.0%)	16 (41.0%)	
NG	7 (10.6%)			
Fecal occult blood				
Positive	40 (60.6%)	24 (60.0%)	16 (40.0%)	0.117
Negative	14 (21.2)%	5 (35.7%)	9 (64.3%)	
NG	12 (18.2%)			

Note: 0.2 is the median of the average methylation ratio; NG: not given.

**Table 2:** Multivariate analysis of prognostic factors for overall survival in CRC patients.

Variables	Hazard ratio	95% CI	*p*
APC2_CPG_14	6.38	1.59–25.64	0.00
Performance status	3.95	1.51–10.29	0.00
Ki67	0.28	0.10–0.77	0.01

## Data Availability

The data used to support the findings of this study are available from the corresponding author upon request.
